# Supplementary figures and images for: Geminivirus C4 proteins inhibit GA signaling via prevention of NbGAI degradation, to promote viral infection and symptom development in N. benthamiana
Source: PLoS Pathog. 2022 Apr 7;18(4):e1010217. doi: 10.1371/journal.ppat.1010217 (PMC9060335; doi:10.1371/journal.ppat.1010217)

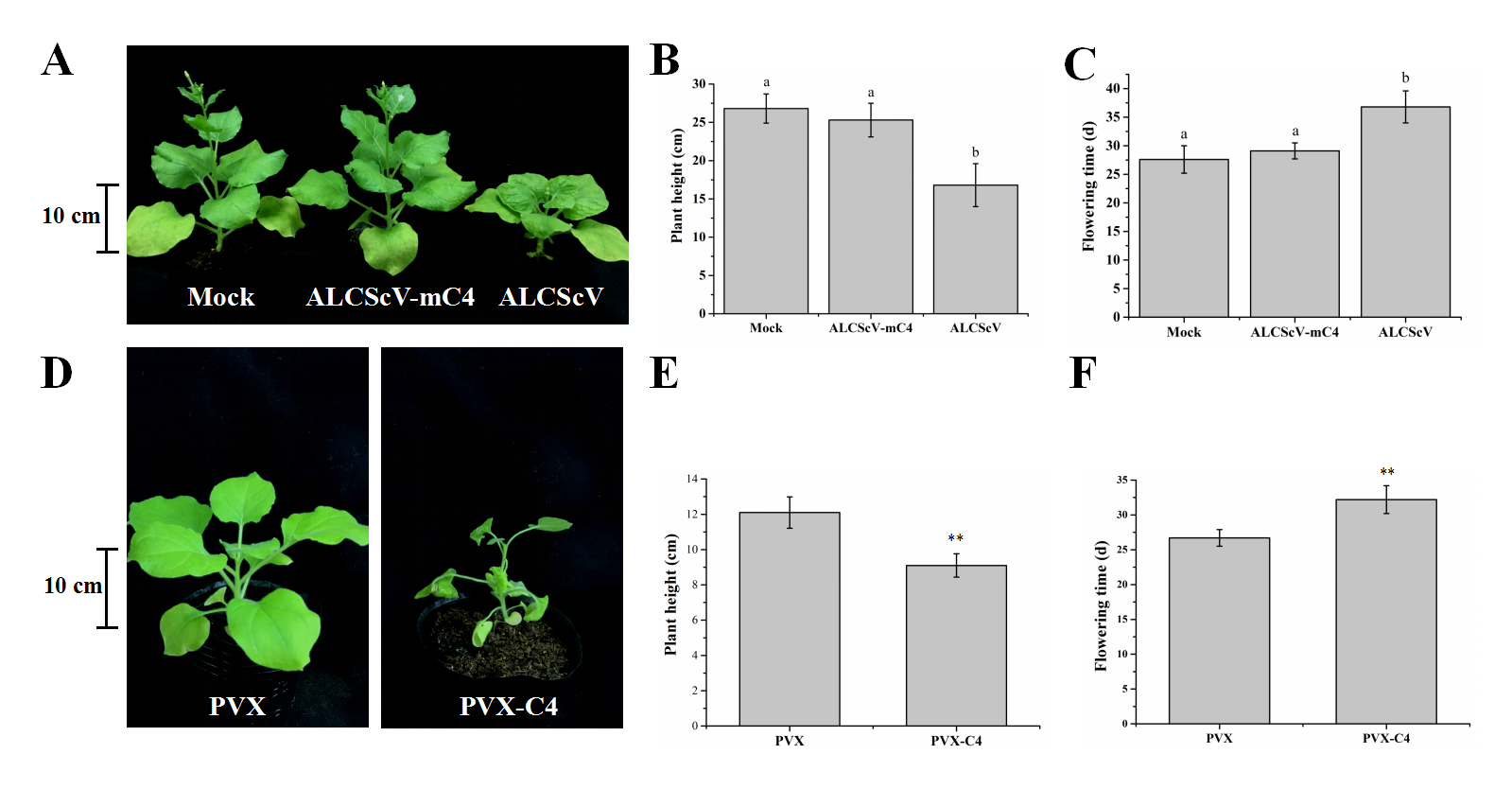

Supplement: S1 Fig — (A) The photographs of the mock-, ALCScV-, and ALCScV-mC4-inoculated N. benthamiana plants at 28 dpi. (B) Results of statistical analysis show the height of the mock-, ALCScV-, and ALCScV-mC4-inoculated N. benthamiana plants at 28 dpi. Different letters above the bars indicate significant differences at the P < 0.05 level. (C) Results of statistical analysis show the flowering time of the mock-, ALCScV-, and ALCScV-mC4-inoculated N. benthamiana plants. (D) The photographs of the PVX- and PVX-C4-infected N. benthamiana plants at 14 dpi. (E) Results of statistical analysis show the height of the PVX- and PVX-C4-inoculated N. benthamiana plants at 14 dpi. (F) Results of statistical analysis show the flowering time of the PVX- and PVX-C4-inoculated N. benthamiana plants. These experiments were performed with three independent biological replicates with similar results. (TIF) [file ppat.1010217.s001.tif]

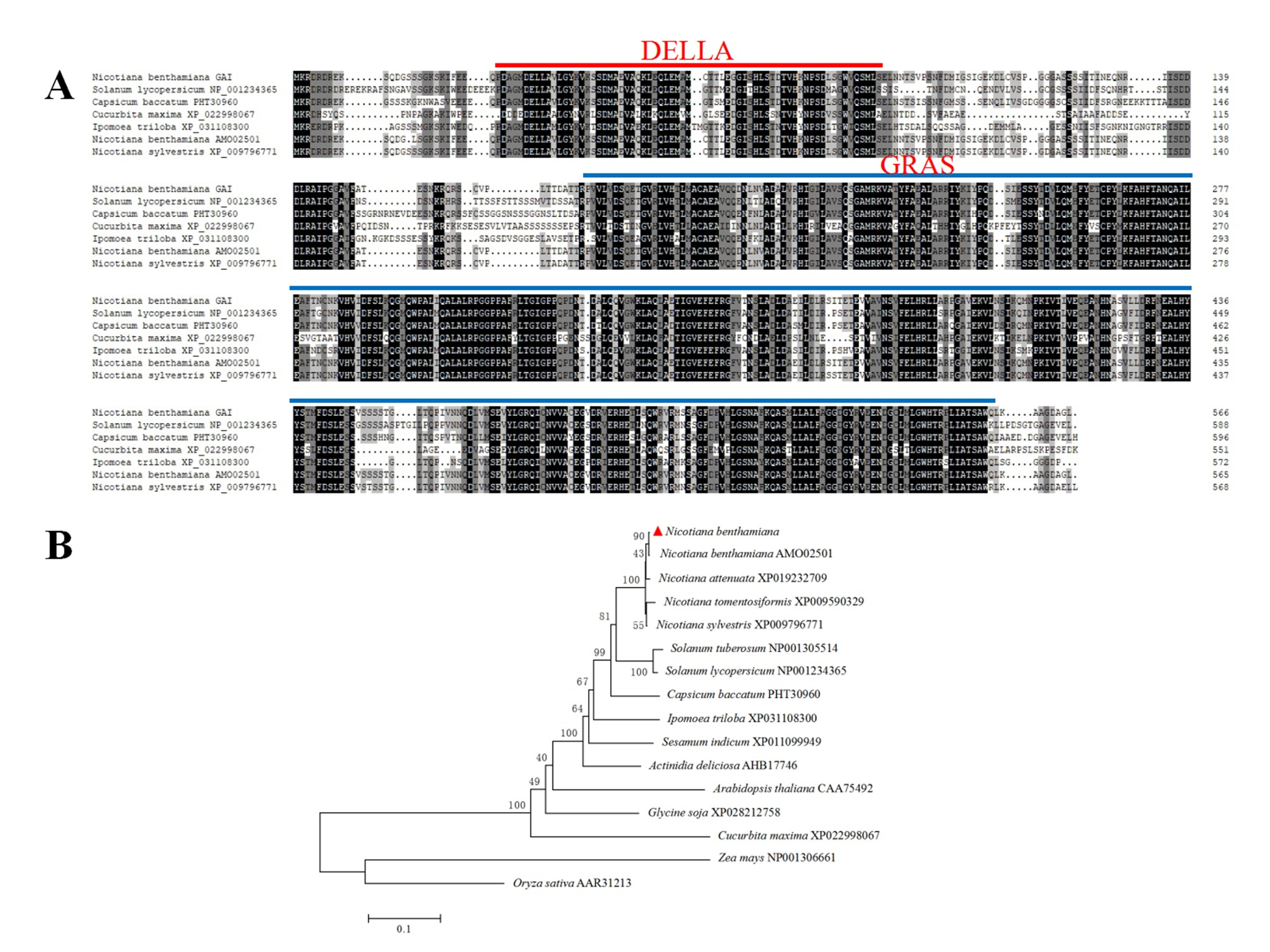

Supplement: S2 Fig — (A) Analysis of conserved domains in the GAI proteins from different plant species. The selected plant species and gene accession numbers are Solanum lycopersicum (NP_001234365), Capsicum baccatum (PHT30960), Cucurbita maxima (XP_022998067), Ipomoea triloba (XP_031108300), Nicotiana benthamiana (AMO02501), and N. sylvestris (XP_009796771). (B) Phylogenetic tree was constructed using the GAI protein sequences from N. benthamiana (AMO02501), N. attenuata (XP_019232709), N. tomentosiformis (XP_009590329), N. sylvestris (XP_009796771), S. tuberosum (NP_001305514), S. lycopersicum (NP_001234365), C. baccatum (PHT30960), I. triloba (XP_031108300), Sesamum indicum (XP_011099949), Actinidia deliciosa (AHB17746), A. thaliana (CAA75492), Glycine soja (XP_028212758), C. maxima (XP_022998067), Zea mays (NP_001306661), and Oryza sativa (AAR31213). The red triangle indicates the GAI protein of N. benthamiana obtained in this study. (TIF) [file ppat.1010217.s002.tif]

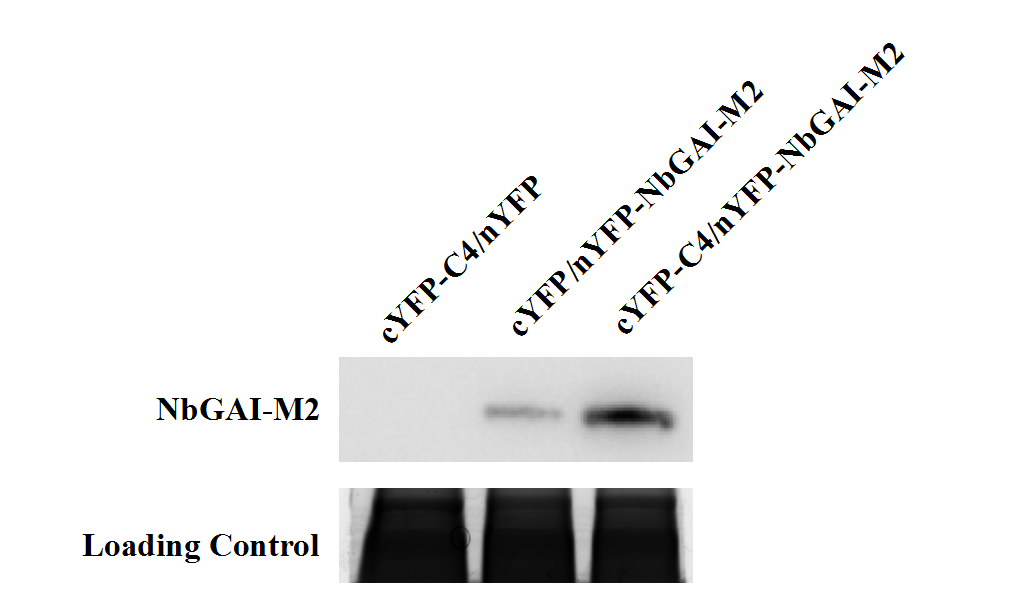

Supplement: S3 Fig — The blot were probed with anti-His antibodies, coomassie brilliant blue (CBB) staining of RuBisCo large subunit gel to confirm equivalent sample loadings. (TIF) [file ppat.1010217.s003.tif]

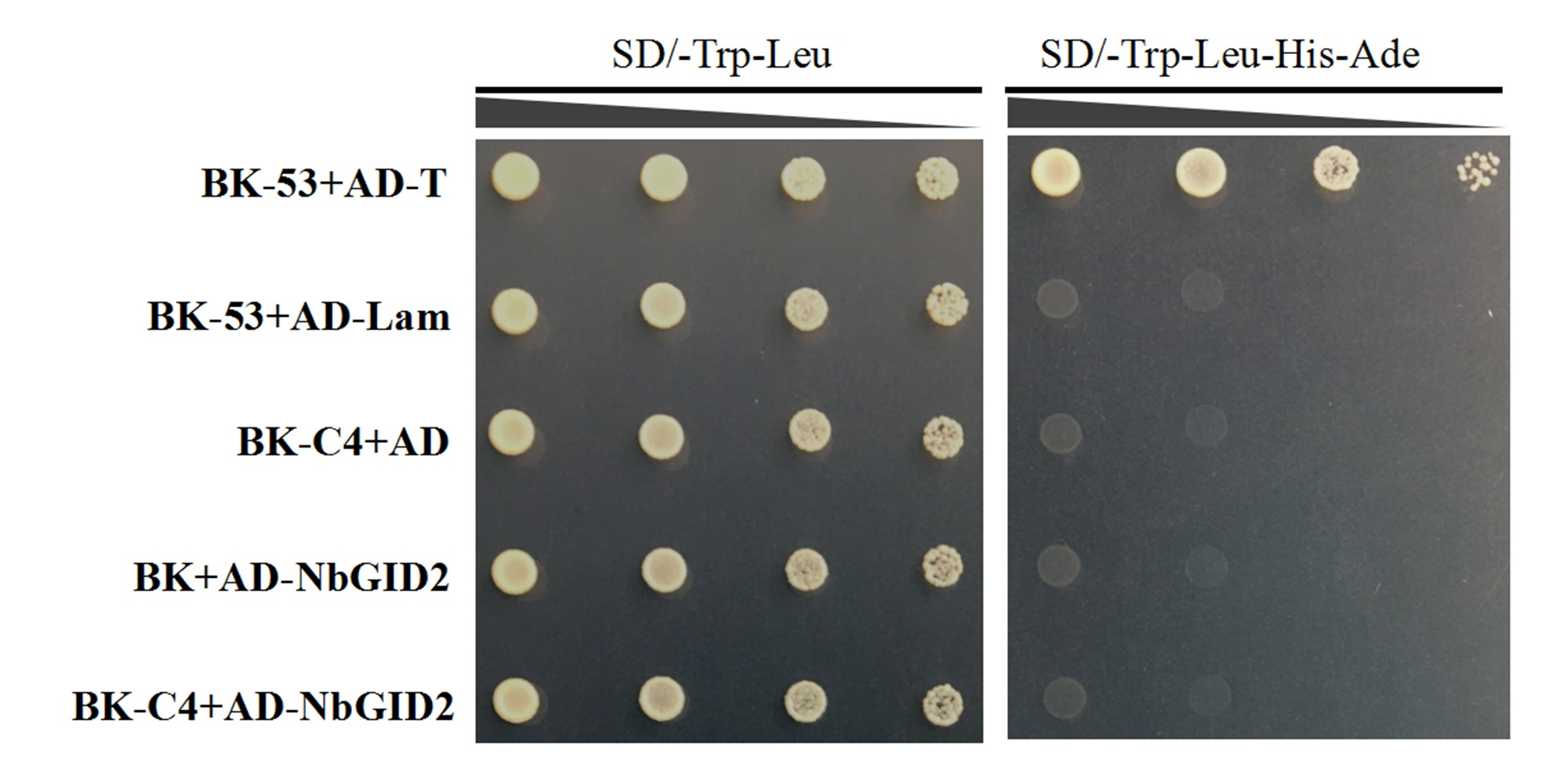

Supplement: S4 Fig — The recombinant plasmids were co-transformed, in various combinations, into S. cerevisiae strain AH109 cells using the lithium acetate method. The transformants were 10-fold serially diluted and then grown on the SD/-Trp/-Leu or the SD/-Trp/-Leu/-His/-Ade medium plates for 3 days. The result show that the C4 does not interact with NbGID2. (TIF) [file ppat.1010217.s004.tif]

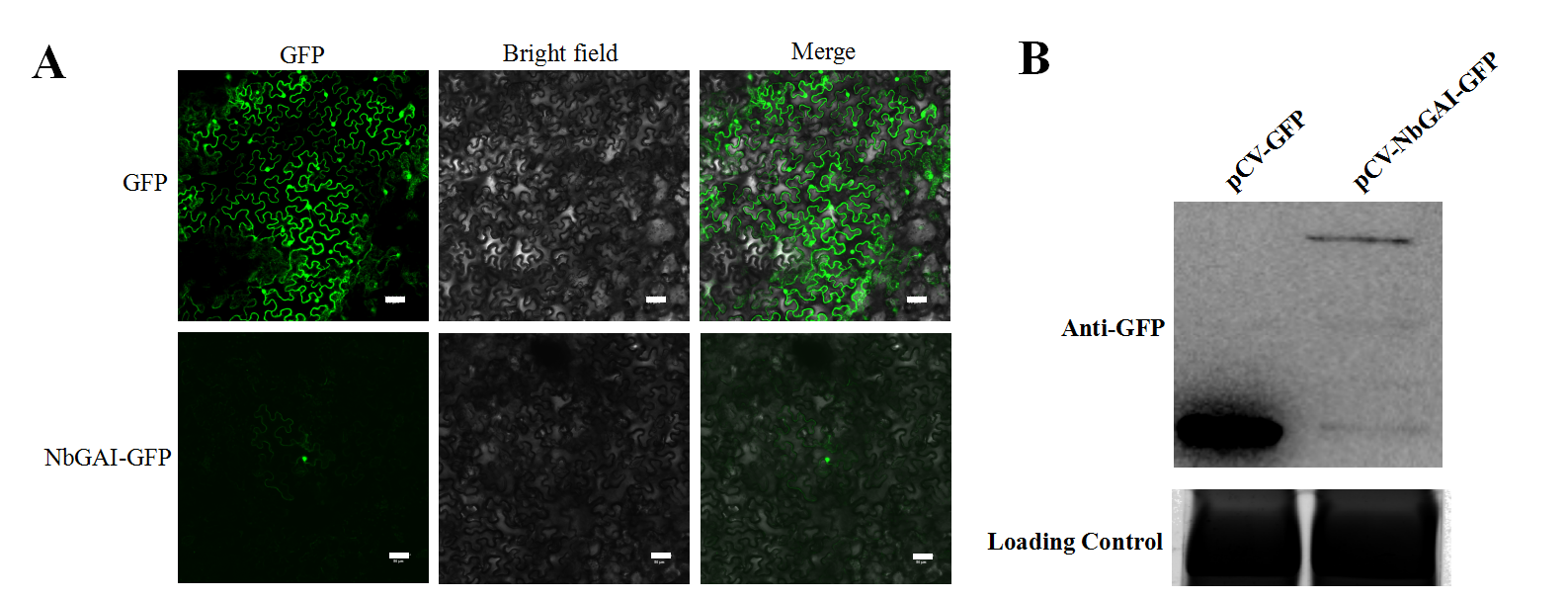

Supplement: S5 Fig — (A) Leaf tissues expressing GFP (top image) or NbGAI-GFP (bottom image) area shown in the left column. Images in the left column were captured under the UV light. The images in the middle column were captured under the bright field. The images in the right column are merged images. Scale bar = 50 μm. (B) Result of Western blot analysis shows the expression level of GFP and NbGAI-GFP fusion protein. The blot was probed with an anti-GFP antibody. The CBB-stained RuBisCo large subunit gel to confirm equivalent sample loadings. (TIF) [file ppat.1010217.s005.tif]

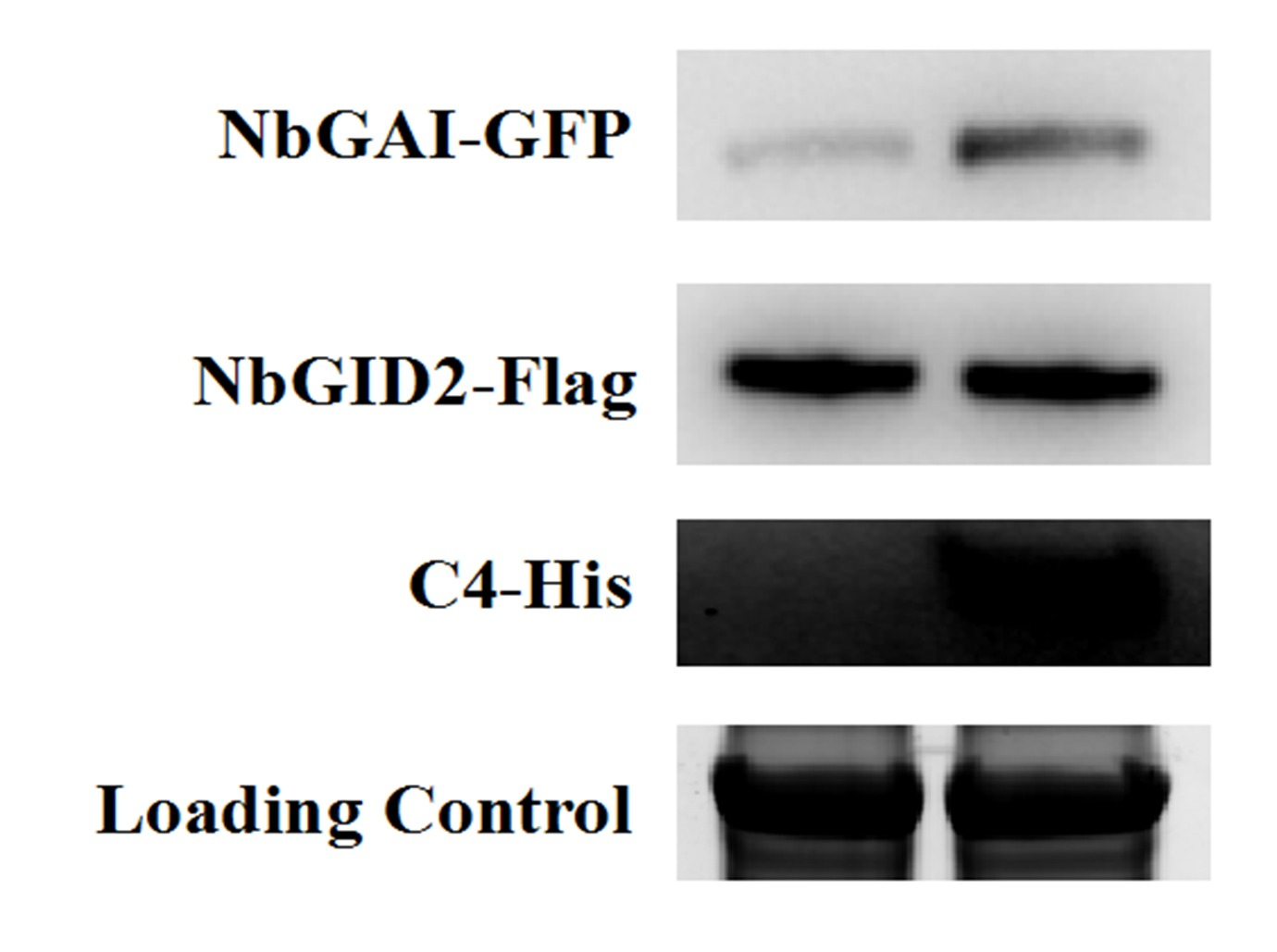

Supplement: S6 Fig — NbGAI-GFP together with NbGID2-Flag were co-expressed with C4-His or the empty vector in N. benthamiana leaf cells, at 2 dpi, the relative accumulation levels of NbGAI-GFP and NbGID2-Flag protein were analyzed by Western blot assay. (TIF) [file ppat.1010217.s006.tif]

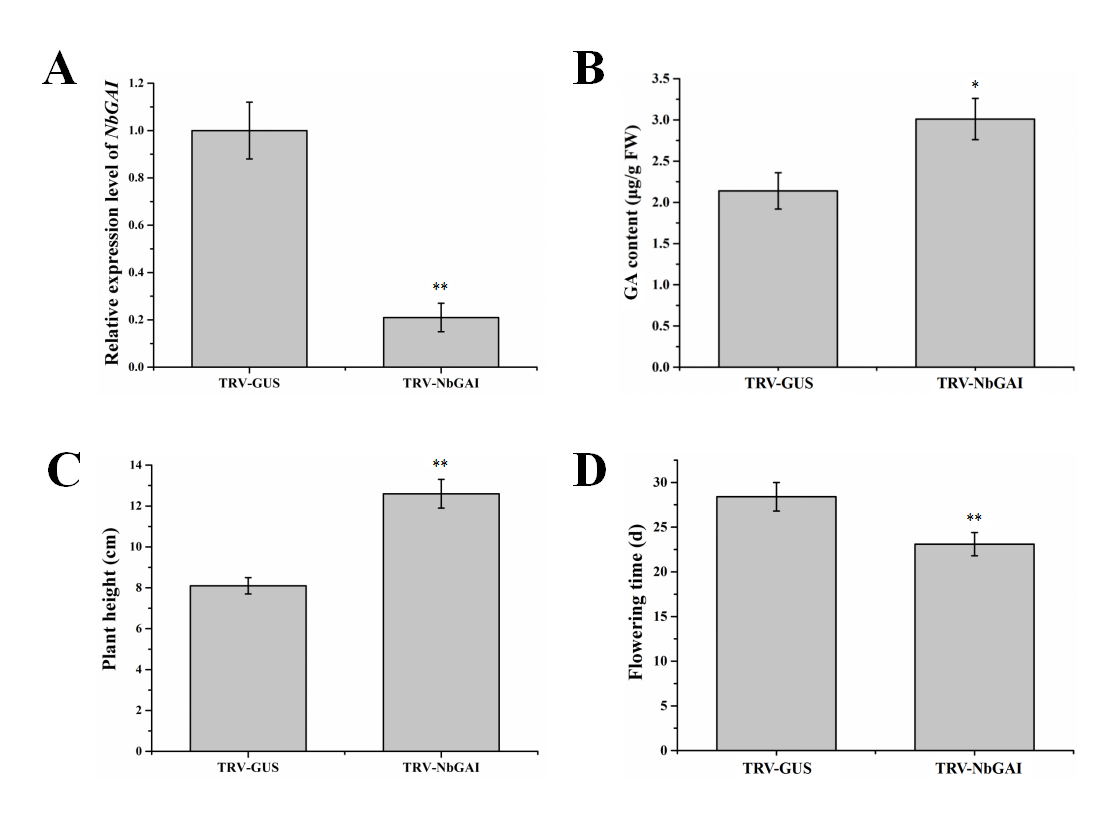

Supplement: S7 Fig — (A) qRT-PCR analysis the relative mRNA levels of NbGAI gene in N. benthamiana plants infected with TRV-GUS and TRV-GAI. At 7 dpi, total RNA were extracted from systemically infected leaves, NbActin gene was selected as internal controls for the assays. All the gene expression data were performed with three biological replicates and three technical replicates, and the relative genes expression levels using the 2-△△Ct method for analysis. (B) The endogenous gibberellin concentrations of TRV-GUS-inoculated and TRV-GAI-inoculated N. benthamiana plants, the leaves were collected at 7 dpi, respectively. (C) Statistical analysis of plant height of TRV-GUS-inoculated and TRV-GAI-inoculated N. benthamiana plants at 14 dpi. (D) Statistical analysis of flowering time of TRV-GUS-inoculated and TRV-GAI-inoculated N. benthamiana plants. “**” indicate an extremely significant difference (P < 0.01 by the Student’s t-test), “*” indicate a significant difference (P < 0.05 by the Student’s t-test). These experiments were performed with three independent biological replicates with similar results. (TIF) [file ppat.1010217.s007.tif]

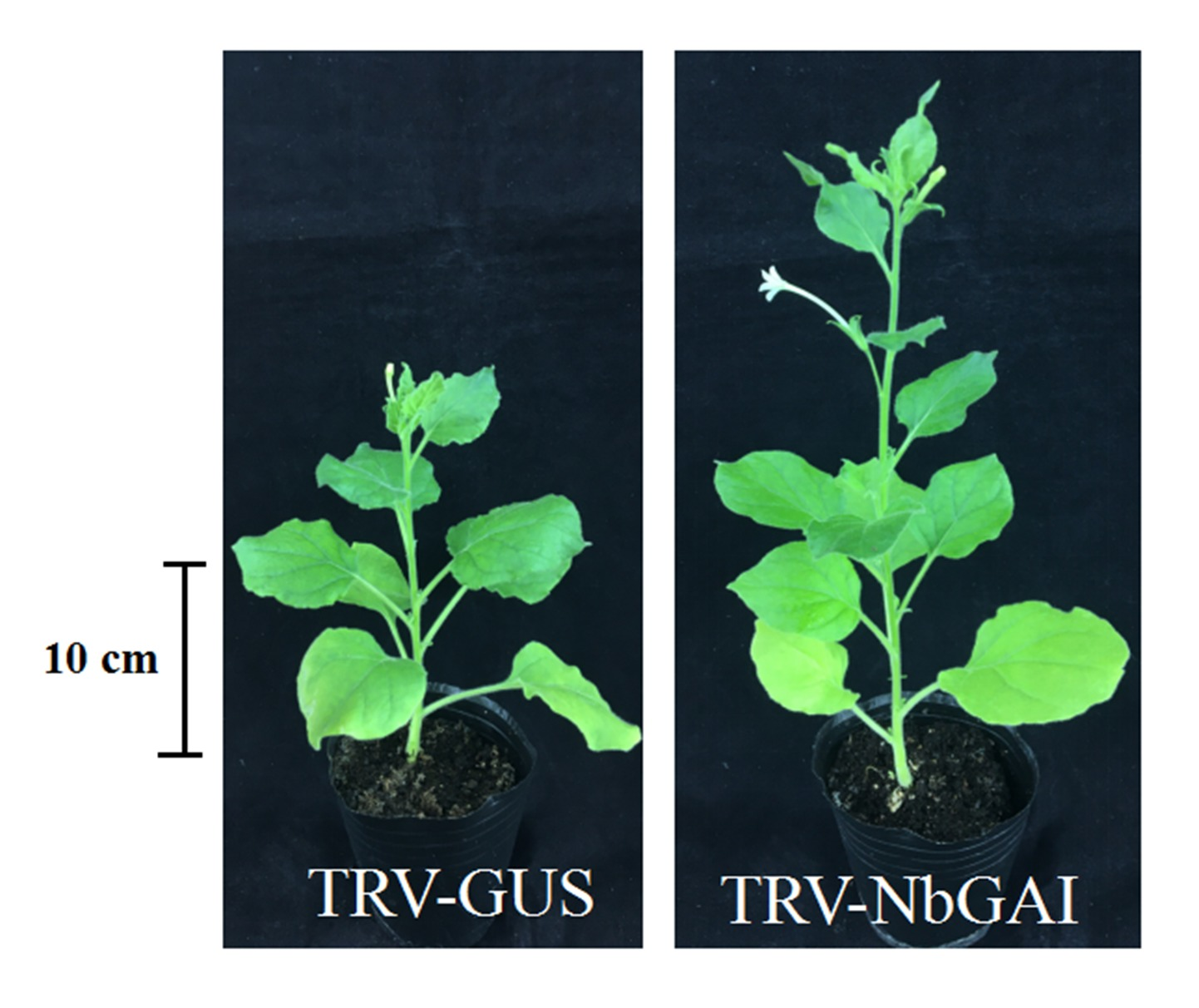

Supplement: S8 Fig — The results show that silencing NbGAI expression in N. benthamiana plants through VIGS significantly increases plant height, but early flowering. The results were reproduced in three independent experiments using 10 plants per treatment. (TIF) [file ppat.1010217.s008.tif]

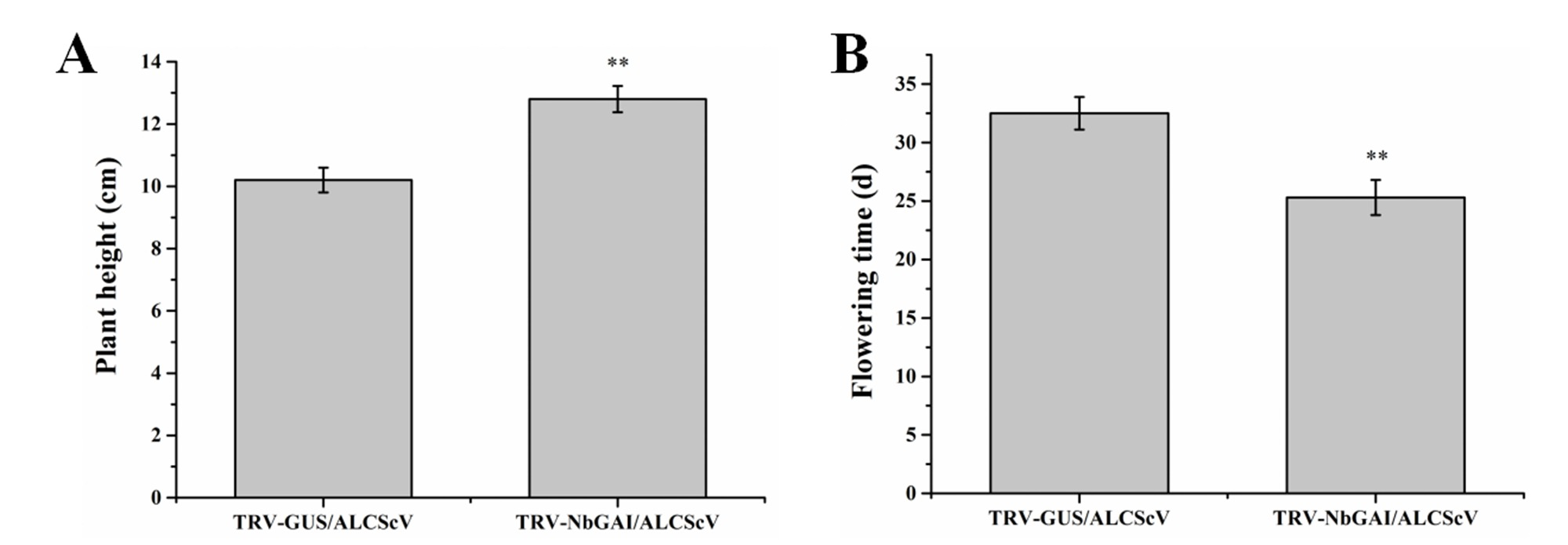

Supplement: S9 Fig — (A) Statistical analysis of plant height of mock-inoculated and ALCScV-inoculated N. benthamiana plants at 14 dpi. (B) Statistical analysis of flowering time of mock-inoculated and ALCScV-inoculated N. benthamiana plants. (TIF) [file ppat.1010217.s009.tif]

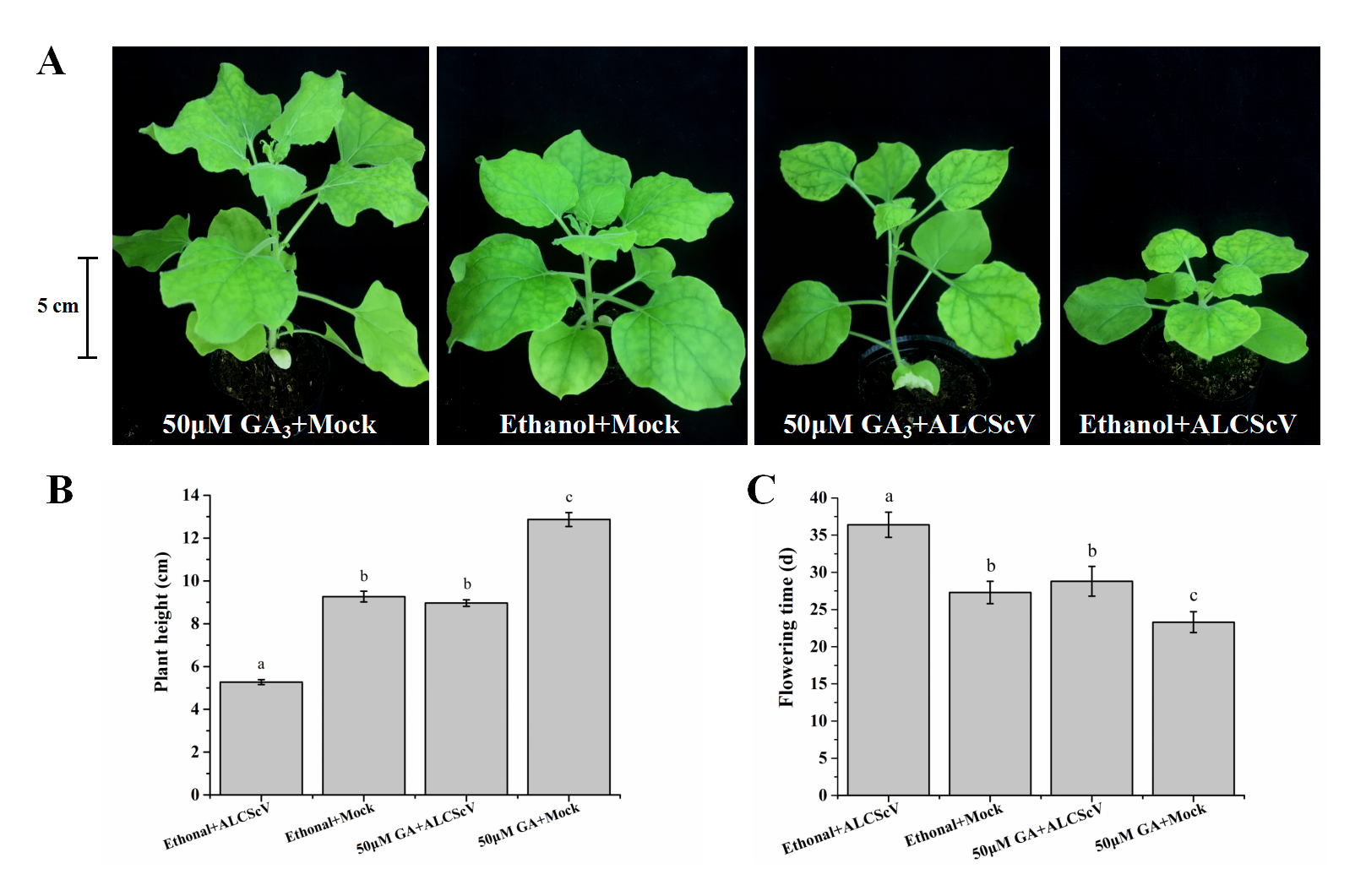

Supplement: S10 Fig — (A) The mock- or ALCScV-inoculated N. benthamiana plants were treated with GA3 or 0.8% ethanol (control), respectively. The plants were photographed at 14 dpi. The results were reproduced in three independent experiments using 10 plants per treatment. (B) Results of statistical analysis show the height of the mock- and ALCScV-inoculated N. benthamiana plants, respectively, at 14 dpi. (C) Results of statistical analysis show the flowering time of the mock- and ALCScV-inoculated N. benthamiana plants, respectively. Different letters above the bars indicate the significant differences among the treatments at the P < 0.05 level. (TIF) [file ppat.1010217.s010.tif]

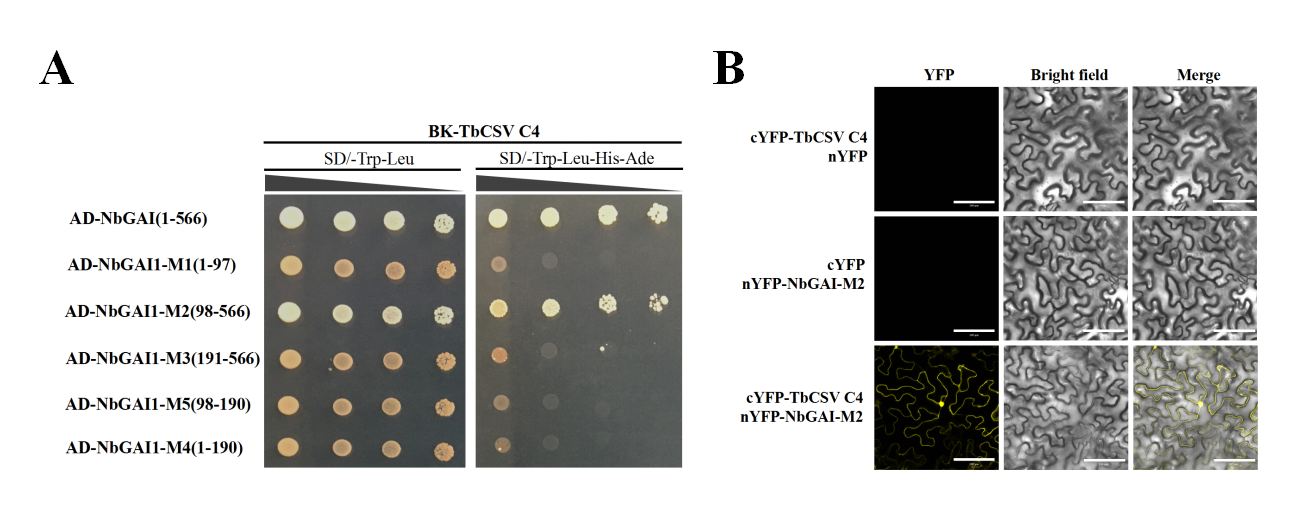

Supplement: S11 Fig — (A) Results of the Y2H assay show that NbGAI-M2 is responsible for the interaction with TbCSV C4. (B) Results of the BiFC assay agrees with the Y2H assay result. (TIF) [file ppat.1010217.s011.tif]

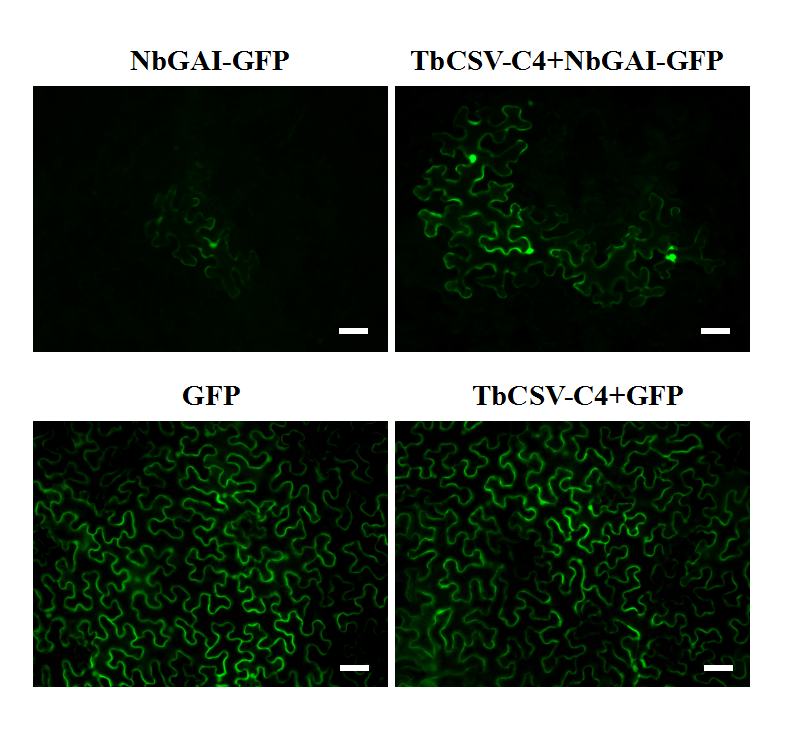

Supplement: S12 Fig — (TIF) [file ppat.1010217.s012.tif]
