# Supplementary material for: Geminivirus C4 proteins inhibit GA signaling via prevention of NbGAI degradation, to promote viral infection and symptom development in N. benthamiana
Source: PLoS Pathog. 2022 Apr 7;18(4):e1010217. doi: 10.1371/journal.ppat.1010217 (PMC9060335; doi:10.1371/journal.ppat.1010217)
Supplement: S1 Table — (DOCX) [file ppat.1010217.s013.docx]

S1 Table. Sequence of primers used in this study

| Primer name | Sequence (5’-3’) |
| --- | --- |
| AD-NbGAI1-F | ATATGGCCATGGAGGCCAGTGAATTCATGAAGAGAGATCGTGATAG |
| AD-NbGAI1-R | ATCTGCAGCTCGAGCTCGATGGATCCCTACAACCCGGCATC |
| AD-NbGAI1-M1-F | ATATGGCCATGGAGGCCAGTGAATTCATGAAGAGAGATCGTGATAG |
| AD-NbGAI1-M1-R | ATCTGCAGCTCGAGCTCGATGGATCCCTAAGTATTGTTGAGCTCAGAT |
| AD-NbGAI1-M2-F | ATATGGCCATGGAGGCCAGTGAATTCATGTCAGTTCCCTCTAATTTC |
| AD-NbGAI1-M2-R | ATCTGCAGCTCGAGCTCGATGGATCCCTACAACCCGGCATC |
| AD-NbGAI1-M3-F | ATATGGCCATGGAGGCCAGTGAATTCATGGCGTGTGCTGAAGCAGT |
| AD-NbGAI1-M3-R | ATCTGCAGCTCGAGCTCGATGGATCCCTACAACCCGGCATC |
| AD-NbGAI1-M4-F | ATATGGCCATGGAGGCCAGTGAATTCATGAAGAGAGATCGTGATAG |
| AD-NbGAI1-M4-R | ATCTGCAGCTCGAGCTCGATGGATCCCTACATTAATGTGTGCACAAG |
| AD-NbGAI1-M5-F | ATATGGCCATGGAGGCCAGTGAATTCATGTCAGTTCCCTCTAATTTC |
| AD-NbGAI1-M5-R | ATCTGCAGCTCGAGCTCGATGGATCCCTACATTAATGTGTGCACAAGT |
| pCV-cYFP-C4-F | GTCGACATGGGAGGCCTCATCTC |
| pCV-cYFP-C4-R | GGATCCCTAGTTCCCTAAGTACTCT |
| pCV-nYFP-NbGAI-F | GTCGACATGAAGAGAGATCGTGATAG |
| pCV-nYFP-NbGAI-R | GGATCCCTACAACCCGGCATCC |
| pCV-nYFP-NbGAI-M2-F | AACATCGAGGACTCCGGAGTCGACATGTCAGTTCCCTCTAATTTC |
| pCV-nYFP-NbGAI-M2-R | GAATTCGAGCTCGCCTGGGGATCCCTACAACCCGGCATC |
| pCV-nYFP-NbGAI-M2-His-F | AACATCGAGGACTCCGGAGTCGACATG*CATCATCACCATCACCAT*TCAGTTCCCTCTAATTTC |
| pCV-nYFP-NbGAI-M2-His-R | GAATTCGAGCTCGCCTGGGGATCCCTACAACCCGGCATC |
| pCV-cYFP-NbGID2-F | GAGCTGTACAAGTCCGGAGTCGACATGAAGCGGCAATTCG |
| pCV-cYFP-NbGID2-R | GAATTCGAGCTCGCCTGGGGATCCTTAAGTTGATTTATTAG |
| pCV-C4-His-F | TCTAGAATGGGAGGCCTCATC |
| pCV-C4-His-R | GGATCCCTAATGGTGATGGTGATGATGGTTCCCTAAGTACT |
| pCV-NbGAI-GFP-F | TCTAGAATGAAGAGAGATCGTGATAG |
| pCV-NbGAI-GFP-R | GTCGACCAACCCGGCATCGC |
| TRV-NbGAI-F | TCGACGACAAGACCCTGCAGGTCCATATCTCAAATTTGCTC |
| TRV-NbGAI-R | TCGACGACAAGACCCTGCAGCATTTGTTTGATCGAATTC |
| BK-NbGID2-Flag-F | TGCATATGGCCATGGAGGCCGAATTCATGAAGCGGCAATTCG |
| BK-NbGID2-Flag-R | TGCGGCCGCTGCAGGTCGACGGATCCTTACTTATCGTCGTCATCCTTGTAATCAGTTGATTTATTAGC |
| BK-NbGAI-GFP-F | TGCATATGGCCATGGAGGCCGAATTCATGAAGAGAGATCGTGATAG |
| BK-NbGAI-GFP-R | TGCGGCCGCTGCAGGTCGACGGATCCTTACTTGTACAGCTCGTC |
| BK-GFP-F | TGCATATGGCCATGGAGGCCGAATTCATGGTGAGCAAGGGCGAG |
| BK-GFP-R | TGCGGCCGCTGCAGGTCGACGGATCCTTACTTGTACAGCTCGTC |
| BK-C4-His-F | TGCATATGGCCATGGAGGCCGAATTCATGGGAGGCCTCATCT |
| BK-C4-His-R | TGCGGCCGCTGCAGGTCGACGGATCCCTAATGGTGATGGTGATGATGGTTCCCTAAGTACT |
| pCV-cYFP-TbCSV C4-F | GAGCTGTACAAGTCCGGAGTCGACATGGGTCTCCTCACCTG |
| pCV-cYFP-TbCSV C4-R | GAATTCGAGCTCGCCTGGGGATCCTTAATATATTGAGGGCCGC |
| BK-TbCSV C4-F | TGCATATGGCCATGGAGGCCGAATTCATGGGTCTCCTCACCTG |
| BK-TbCSV C4-R | TGCGGCCGCTGCAGGTCGACGGATCCTTAATATATTGAGGGCCGC |
| NbGAI-q-F | CGACATGATTGGTAGTATTGG |
| NbGAI-q-R | CCTTGTTGTTGCATCTGTT |
| NbGID2-q-F | CGGCTCTACTCGCTTTATC |
| NbGID2-q-R | ACTTCATCTTTTCCCCATC |
| NbGA20-q-F | AGGCGATACATTTATGGCTCTTTC |
| NbGA20-q-R | GCTCACTACCTTATCCTTCTTTGG |
| NbGA-3β-q-F | CATTGTTGGCTCCCCTCTTG |
| NbGA-3β-q-R | TTCCATTTCCCTTTCGTATTCTTC |
| NbKS-q-F | TCTGATGTTGGAGGTTCTATGG |
| NbKS-q-R | CACTTAAATGCCTCATCTCC |
| NbGID1B-q-F | TCTGTCGTCGCCTTGTTAGCAT |
| NbGID1B-q-R | AGAGCACTCCATCCATCATC |
| NbGID1C-q-F | ATGGACTGCTCTTGAGTGGGT |
| NbGID1C-q-R | CTGCCCTGAAAGCCACATTGT |
| NbPIF4-q-F | TGGTTCAAGTCACTGTGGTAG |
| NbPIF4-q-R | GTCCATTGTATCACTTTGCG |
| NbPIF3-q-F | GGTTCAAAGAGAAGCCGAGCT |
| NbPIF3-q-R | AAGCTTTATCCGCCTTGTTGC |
| ALCScV-qPCR-F | CCCGTGTATGCTACTTTGAAAG |
| ALCScV-qPCR-R | ACCTCACACCTTCAGACTGG |
| TbCSV-qPCR-F | GGTCCATGTAAGGTCCAGTC |
| TbCSV-qPCR-R | GATGAGTCAGCCCAGTTCC |
| Nb25SRqF | GCGAGTAAACCCGTAAGG |
| Nb25SRqR | GCTCAGGCATAGTTCACC |
