# Supplementary material for: Geminivirus C4 proteins inhibit GA signaling via prevention of NbGAI degradation, to promote viral infection and symptom development in N. benthamiana
Source: PLoS Pathog. 2022 Apr 7;18(4):e1010217. doi: 10.1371/journal.ppat.1010217 (PMC9060335; doi:10.1371/journal.ppat.1010217)
Supplement: Attachment — Submitted filename: Response to reviewers.doc [file ppat.1010217.s015.doc]

10^th^ March, 2022

Dear editor,

Many thanks for reviewing our manuscript. We have revised the text accordingly to the comments and recommendations by the referees and editor. As requested, a point-by-point response to the reviewer’s comments was listed out as following. The major changes were marked in red color in revised manuscript.

Thank you very much for your help,

Sincerely,

Ling Qing

College of Plant Protection

Southwest University

Chongqing 400716

P. R. China

[qling@swu.edu.cn](mailto:qling@swu.edu.cn)

**Responds to the reviewer’s comments:**

**Major Issues: Key Experiments Required for Acceptance**

**Reviewer #1:**

1 The protein expression of GAI-M2 in Fig 3 should be confirmed by western blot.

OUR RESPONSE: Thank you for your suggestion. The protein expression of NbGAI-M2 was confirmed by western blot which was added in S3 Fig.

2 It is known that the interaction between GAI and GID2 can enhance GAI, degradation via the ubiquitin-26S proteasome. In this study, to test whether ALCScV C4 could also affect the stability of NbGAI, NbGAI-GFP was transiently expressed in *N. benthamiana* leaves through agro-infiltration, NbGAI-GFP was co-expressed with C4-His or the empty vector in *N. benthamiana* leaf cells. Authors should analyze “NbGAI-GFP together with GID2-tag was co-expressed with C4-His or the empty vector in *N. benthamiana* leaf cells”, to confirm whether ALCScV C4 could affect the stability of NbGAI and NbGID2.

OUR RESPONSE: Thank you for your suggestion. According to your suggestion, NbGAI-GFP together with NbGID2-Flag were co-expressed with C4-His or the empty vector in *N. benthamiana* leaf cells, at 2 dpi, the relative accumulation levels of NbGAI-GFP and NbGID2-Flag protein were analyzed by Western blot assay. The results showed that C4 protein can inhibit the degradation of NbGAI protein, but not affect the stability of the NbGID2 protein (It has been added in S6 Fig).

3 GAs are essential for many developmental processes in plants. Therefore, it is reasonable that C4 interferes with GAI to induce abnormal development. However, how GAs influence viral DNA accumulations? Which resistance pathway is involved in this? The authors should discuss it.

OUR RESPONSE: Thank you for your questions and suggestion. Crosstalk between plant hormones is the core of plant stress response. GA does not work independently but acts in a complex signaling network combined with other plant hormone signaling pathways. For example, previous work showed that DELLAs regulate immunity by modulating the balance between the archetypal defense hormones SA and JA. Because treatment of plants with exogenous GAs can promote the degradation of DELLAs via the ubiquitin-26S proteasome, therefore, we speculate that exogenous application of GAs may disrupt the balance of SA and JA through the degradation of the DELLAs protein, thereby affecting virus infection. Meanwhile, it has been shown that DELLAs can interact with BZR1, leading to the inhibition of DNA-binding ability of BZR1, and the GA-induced DELLA degradation enhances BR signaling. BR has been shown to play a key role in coping with viral infections. For example, exogenous application of BR has been shown to alleviate TYLCV-caused symptoms in tomato and BCTV C4-induced developmental abnormalities in *Arabidopsis*. In addition, GAs and DELLAs contribute to fine-tuning of ROS production, among them, GA can induce oxidative stress in plants, and can lead to programmed cell death, and contributes to enhanced disease resistance.

Based on these studies, therefore, we speculate that GAs influence viral DNA accumulations through crosstalk with other plant endogenous hormones (such as SA, JA, and BR) or by regulating ROS production. It has been added in discussion (Pages 22 and 23, lines 534-554).

**Reviewer #2:**

Biological replicates are needed – and should be indicated – for all experiments. Having three plants per experiment (as in the case of the exogenous GA treatment presented in Figure 10) is not sufficient and does not provide an idea of the reproducibility of the results.

OUR RESPONSE: Thank you for your suggestions. It could be a misunderstanding caused by our unclear expression in the manuscript. In this study, all experiments were repeated three times. For the inoculation experiment and the GA treatment experiment, ten biological replicates for each treatment and three independent replicates experiments were carried out respectively. In the manuscript, we described that the experiment had at least three biological replicates (plants) per treatment, this description may not be accurate, it has been revised accordingly (Lines 164-165, 394-395, 418-419, 447-448, 959-960, 969-970).

The quality of the confocal images is in general not sufficient.

OUR RESPONSE: Thank you for your suggestion. It has been revised accordingly (Fig 2B, Fig 3C, Fig 4B, Fig 4C, Fig 4F, Fig 5A, Fig 6A, Fig 6E, Fig 10B, S11B Fig).

English needs editing throughout the manuscript.

OUR RESPONSE: Thank you for your suggestion. Our manuscript has been edited and revised by professional language editors as shown in the full text.

Multiple references are missing in the introduction. The authors can check, for example, the recent review by Devendran et al. (2022) on geminivirus-encoded proteins, or the C4-specific review by Medina-Puche et al. (2021).

OUR RESPONSE: Thank you for your suggestion. It has been added in the introduction, with reference numbers 11 and 12 (Page 4, lines 66-69; Page 30, lines 730-735).

**Minor Issues: Editorial and Data Presentation Modifications**

**Reviewer #1:**

1 Lack scale bars in the picture with plants, Fig 1A, 8A, 8F, 9A, 10C.

OUR RESPONSE: Thank you for your suggestion. The scale bars were added in Fig 1A, 8A, 8B, 9A, 10C.

2 Scale bars in con-focal images are too small and too obscure to read.

OUR RESPONSE: Thank you for your suggestion. It has been revised accordingly (Fig 2B, Fig 3C, Fig 4B, Fig 4C, Fig 4F, Fig 5A, Fig 6A, Fig 6E, Fig 10B, S11B Fig, S12 Fig).

3 Clear and amplified con-focal images should be replaced with the current images, which are hardly to see the details of their subcellular localisations in the cell.

OUR RESPONSE: Thank you for your suggestion. Clear and amplified con-focal images have replaced current image.

4 Fig 8-B-C-D-E should be incorporated into one figure.

OUR RESPONSE: Thank you for your suggestion. It has been revised accordingly (It has been added in S7 Fig) .

5 There should be a space between number and dpi.

OUR RESPONSE: Thank you for your suggestion. It has been revised accordingly.

6 Line 483 Rice Line 83, 506 Cotton.

OUR RESPONSE: Thank you for your suggestions. It has been revised accordingly (Lines 484, 506).

**Reviewer #2:**

- In Figure 10, how was the treatment performed? Repeatedly, or only once? The effect of the hormone treatment of the ability of *Agrobacterium tumefaciens* to transform plant cells should be tested.

OUR RESPONSE: Thank you so much for your questions. In Figure 10, for GA_3_ treatment assays, *N. benthamiana* plants at the 4-6-leaf stage were sprayed with a solution containing 50 μM GA_3_ or 0.8% ethanol (mock treatment). After 12 h, the sprayed plants were inoculated with ALCScV through agroinfiltration method. The inoculated plants were grown inside the growth chamber and sprayed with the 50 μM GA_3_ solution or the 0.8% ethanol solution once every other day till 7 dpi (a total of four spraying was performed). It is described in the materials and methods of the manuscript (Pages 27 and 28, lines 654-661).

In Fig 6E and 6F, pCV-GFP was transiently expressed in *N. benthamiana* leaves through agro-infiltration. However, whether plants were treated with 50 μM GA_3_ or the 0.8% ethanol solution (mock treatment), the green fluorescence was not significantly different. Meanwhile, the results of Western blot assay showed that the accumulation level of GFP protein was not significantly different between 50 μM GA_3_ or the 0.8% ethanol solution treatments. These results revealed that hormone treatment has no effect on the ability of *Agrobacterium tumefaciens* to transform plant cells.

- The authors should discuss the GA/BR interplay. This is particularly relevant in the case of this work, since C4 from other geminiviruses has been shown to affect BR signaling, and exogenous application of BR has been shown to alleviate TYLCV-caused symptoms in tomato (Seo et al., 2018) and BCTV C4-induced developmental abnormalities in *Arabidopsis* (Mills-Lujan and Deom, 2010).

OUR RESPONSE: Thank you for your suggestions. We have added the discussion about the GA/BR interplay on page 23, lines 542-549.

- Please check the spelling of “ethanol” in Figure 9.

OUR RESPONSE: Thank you for your suggestion. It has been checked and revised accordingly.

- Please follow the ICTV guidelines on how to write virus names.

OUR RESPONSE: Thank you for your suggestion. We have revised virus names accordingly followed the ICTV guidelines. The article that we referenced was “Differentiating between viruses and virus species by writing their names correctly”.

Finally, thank you again for your positive comments and valuable suggestions to improve the quality of our manuscript.
